# 

**Published:** 1915-07

**Authors:** 


ATLANTA, GA.

PUBLISHED MONTHLY. SUBSCRIPTION $1.00

### ATLANTA JOURNAL-RECORD OF MEDICINE

Successor to { Atlanta Medical and Surgical Journal, Established 1855. and Southern Medical Record, Established 1870. } 62nd Year

Vol. LXII ATLANTA, GA., JULY, 1915. No. 4

